# Spatial variations and determinants of anemia among under-five children in Ethiopia, EDHS 2005–2016

**DOI:** 10.1371/journal.pone.0249412

**Published:** 2021-04-01

**Authors:** Zelalem Alamrew Anteneh, Jean-Pierre Van Geertruyden

**Affiliations:** 1 Department of Epidemiology, Bahir Dar University, Bahir Dar, Ethiopia; 2 Global Health Institute, University of Antwerp, Antwerp, Belgium; Helen Keller International, UNITED KINGDOM

## Abstract

**Background:**

Anemia has severe public health significance in sub-Saharan Africa. In Ethiopia, anemia has been increasing in the last two decades, reaching the highest national level in 2016, however, the geospatial distribution and determinants of anemia in children weren’t well explored at a national level.

**Methods:**

We used the Ethiopian Demographic and Health Survey(EDHS) data from 2005–2016. The data consists of samples of households (HHs) obtained through a two-stage stratified sampling procedure. Our analysis included 19,699 children. Descriptive statistics, geospatial analysis, and Generalized Linear Mixed Model (GLMMs) were used.

**Results:**

The overall prevalence of anemia was 51.5%; the spatial distribution of anemia significantly different across clusters in each survey. Children from 6 to 11 months had higher odds of anemia compared to 24–59 months (Adjusted Odds ratio (AOR) = 3.4, 95%Confidence level (CI): 2.99–3.76). Children with the first and second birth order were less likely to be anemic compared to fifth and above (AOR = 0.60, 95%CI: 0.38–0.95, and AOR = 0.83, 95%C: 0.73–0.93) respectively. Mothers’ age 15 to 24 years was associated with higher odds of anemia compared to 35 to 49 years (AOR = 1.37, 95%CI: 1.20–1.55). Children from HHs with the poorest and poorer wealth category showed a higher odds of anemia compared to the richest (AOR = 1.67, 95%CI: 1.45–1.93, and AOR = 1.25, 95%CI: 1.08–1.45) respectively. Moreover, children from HHs with one to two under-five children were less likely to be anemic compared to those three and more (AOR = 0.83, 95%CI: 0.76–0.91).

**Conclusions:**

The geospatial distribution of anemia among children varies in Ethiopia; it was highest in the East, Northeast, and Western regions of the country. Several factors were associated with anemia; therefore, interventions targeting the hotspots areas and specific determinant factors should be implemented by the concerned bodies to reduce the consequences of anemia on the generation.

## Introduction

Anemia is a condition in which the ability of red blood cells to carry oxygen is impaired, insufficient to meet the physiologic needs of the body [1, 2]. It is an indicator of both poor nutrition and health.

According to the global burden of disease report in 2016, anemia affects more than 27% of the world’s population, nearly, 1.93 billion people. Low- and middle-income countries account for more than 89% of the cases. Preschool children and reproductive-aged women are disproportionately affected by anemia [3]. In 2017, the World Health Organization (WHO) data repository showed that the global prevalence of anemia in under-five children was 41.7%. The problem is worse in the WHO Africa region, 59.3% of children under five were anemic [4]. In Ethiopia, the prevalence of anemia in children less than 5 years is persistently higher than expected for the last two decades [5]. In Sub-Saharan African countries, anemia is one of the major challenges to health affecting more than 40% of children and is considered a severe public health significance in the region [6]. This demands to investigate the component causes of anemia to implement the corresponding intervention strategies.

Iron deficiency is the major cause of anemia, but there are several other causes including folate, vitamin B12, and vitamin A deficiencies, and chronic inflammation, parasitic infections, and inherited disorders [2]. Micronutrients are often associated with specific physiological processes in the body; deficiency at an early age can affect children’s cognitive and motor development [7, 8].

Studies showed that several factors have been affecting the occurrence of anemia in children, a systematic review study indicated that the distribution of anemia is more prevalent among children from rural communities compared to urban ones [9]. Similarly, a study conducted in Bangladesh revealed that children from rural communities were at higher risk of anemia compared to children from urban communities [10]. Evidence is showing younger children are at higher risk of anemia compared to older ones; studies conducted in Uganda and Ghana indicated that children less than 24 months of age had a serious risk compared to older children [11, 12].

Studies also pointed out that maternal anemia affects the anemia status of children [13, 14]. Besides, household wealth status, family size, and the number of children in the household influenced childhood anemia [11, 15]. Regardless of the persistent higher magnitude of childhood anemia among children in Ethiopia across the waves of EDHS, spatial distributions, and determinants of anemia in children were not well explored at a national level. Studying regional variations and determinants of anemia has an important policy implication to evaluate the progress being made by the regional states in the country. In addition, studying the geospatial variations of anemia helps to identify the most hotspot areas of the problem. This helps to inform the government where to allocate its scarce resource for implementation of interventions to minimize the effect of anemia on the generation.

## Methods and materials

### Study design and setting

This study used data from nationally representative, cross-sectional Ethiopian Demographic, and Health Survey (EDHS) conducted from 2005 to 2016 [5, 16, 17].

Administratively, Ethiopia has nine regional states namely; Amhara, Oromia, Tigray, Benishangul-Gumuz, Somali, Afar, Harari, Southern Nations Nationalities and Peoples (SNNP), Gambella, and two city administration councils (Addis Ababa and Dire Dawa. The regions are subdivided into Zones, each Zone into Woredas, and Woreda into the lowest administrative units named Kebeles. For sampling purposes, each kebele was subdivided into census enumeration areas (EAs) [18].

### Sampling strategy

EDHS consist of a sample of HHs obtained through a two-stage stratified sampling procedure. The EDHS uses the Ethiopia Population and Housing Census (PHC) sampling frame prepared by the Ethiopia Central Statistical Agency (CSA). Samples were selected using a stratified, two-stage cluster sampling design, where enumeration areas (EAs) were the primary sampling units for the first stage, & house-holds (HHs) for the second stage. Representative samples of 32,156 (9,861 in 2005, 11,654 in 2011, and 10,641 in 2016) under-five children were included in the respective surveys. The EDHS collects blood samples among all children of age 6 to 59 months included in the survey for hemoglobin tests. A total of 19,699 (3394 in 2005, 8510 in 2011, and 7795 in 2016) children gave blood specimens for hemoglobin tests; therefore, we included them in our analysis.

### Outcome measurements

Blood specimens were collected from 6–59 months children for whom consent was obtained from their parents or caregivers responsible for them. Blood samples were drawn from a drop of blood taken from a finger prick (or a heel prick in the case of children age 6–11 months) and collected in a microcuvette. Hemoglobin analysis was carried out on-site using a battery-operated portable HemoCue analyzer. The hemoglobin values obtained using the HemoCue instrument were adjusted for altitude before classification into the level of anemia. Based on the WHO hemoglobin level cut off points, the hemoglobin from 10.0–10.9 g/dl is mild, 7.0–9.9 g/dl is moderate, and level less than 7.0 g/dl severe anemia. Therefore, the hemoglobin level less than 11 g/dl of blood is anemic, otherwise normal.

### Independent variables

Potential determinant factors of anemia in children were extracted from the EDHS dataset. The factors were selected based on previous studies [10, 15, 19–21], and our knowledge. These factors were categorized in the following ways:

**Individual-level factors**. Sex, age, size at birth, stunting, wasting, underweight, birth order, fever, diarrhea, cough, child twin status, birth intervals, mother’s and father’s educational level, age of mother, marital status, mothers’ working status, religion, mother’s media exposure.
**Household and contextual level factors**. Residence (urban, rural), source of drinking water, type of toilet facility, type of cooking fuel, wealth index, number of children U5 in the family, number of household size.

### Operational definitions

Most of the determinant factors in our analysis were used as they are in their original coding from EDHS; however, some variables were created by combining two or more variables or regrouping the levels of the variables.

Sources of drinking water: the source of drinking water has several levels in the original coding, however, we regrouped into (improved and unimproved water sources), and Toilet facility: toilet facility consisted of several levels in the original coding and regrouped into (improved and unimproved toilet facility) based on WHO classifications [22]. Similarly, cooking fuel was grouped into (Electricity and gas, fossil fuels, charcoal, and agricultural products & animal dung) [23], and the presence of media exposure into “Yes” (women who listen to the radio, watch TV, or reads magazines at least once in a week), otherwise “No” [5].

#### Birth size

Children whose birth weight is less than 2.5 kgs, or children reported to be ‘very small’ or ‘smaller than average’ are considered to low birth weight, otherwise normal. According to the EDHS sources, birth size (birth weight) was collected based on the presence of written records if available, or based on the mother’s report [5, 16, 17].

#### Stunting

Height-for-age less than minus two standard deviations (-2SD) from the median of the reference population were regarded as moderately stunted, while below −3SD from the median of the reference population were considered severely stunted.

#### Underweight

Weight-for-age less than -2SD from the median of the reference population were regarded as moderately underweight, while below −3SD from the median of the reference population were considered severely underweight.

#### Wasted

Weight-for-height less than -2SD from the median of the reference population were regarded as moderately wasted, while below −3SD from the median of the reference population were considered severely wasted. These anthropometric indicators were measured based on the WHO growth standard [24].

#### Wealth index

Wealth index is a measure of the socioeconomic status of the households to indicate inequalities in society. The households were given scores based on assets (television, bicycle/car, size of agricultural land, the quantity of livestock), and dwelling characteristics (sources of drinking water, sanitation facilities, and materials used for constructing houses) using principal component analysis, and the scores were compiled into five categories of wealth quintile (poorest, poorer, medium, richer and richest) each comprising 20% of the population [5, 16].

### Spatial analysis

Spatial analysis was done using the application of Geographic Information System (GIS) to determine geographic variations of anemia cases among EDHS clusters for each wave from 2005 to 2016. ArcGIS software version 10.1 was used. We received the GPS points in shapefile format for each EDHS survey from the DHS office upon request. We computed the proportions of anemia cases for each cluster in each survey. We downloaded the images of administrative boundaries of Ethiopia in shapefile form from DHS website [25]. Then, we appended the proportions of anemia with the shapefile of the clusters. The high and low hotspots of anemia were visualized for each cluster in each survey. The Getis-Ord G-statistic was used to show the overall patterns of high/low clustering of anemia among children in enumeration areas.

Spatial variations of significantly high and low hotspots of anemia were computed for each cluster in each survey using the Getis-Ord G* statistic tool. The clustering of statistically high hotspots of percentages of anemia is shown by a positive z-score with a P-value of <0.05, however, clustering of statistically low spots of anemia is indicated by a negative z-score with a p-value of <0.05. A z-score near zero indicates no apparent clustering.

### Statistical analysis

Descriptive statistics such as frequency distributions for respondents and children under five years were done. The prevalence of anemia in under-five children by different backgrounds and contextual characteristics of the HHs was computed. In addition, the prevalence of anemia among under-five children across the waves of EDHS 2005 to 2016 was computed for whole regions of Ethiopia. We used weighted data analysis to account for the difference in the sampling proportions to avoid distortions in our estimates. STATA version 13 and SPSS Version 25 software packages were used for analysis. Bivariate logistic regression analysis was used to identify potential candidate independent factors associated with anemia among under-five children. Variables that were associated with anemia at a p-value of 0.20 level of significance were selected to enter multilevel logistic regression models.

A generalized linear mixed model (GLMMs) was carried out to examine the effects of individual, and household, and contextual factors on childhood anemia. We fit three phases of modeling. The first (Model 1) was the null model with no individual or household level factors. It consisted of only cluster-specific random effects to model between-cluster variations in anemia. The second model (Model II) incorporated individual-level factors in addition to cluster-specific random effects. The third model (Model III) contained both individual, and household, and contextual factors in addition to cluster-specific random effects. For the fixed part of the model, the results were presented with ß-coefficients, standard errors, p-values, and OR with its 95% confidence level, however, for the random part, variance estimate with its standard error and 95% confidence level was used to present the results.

Multicollinearity tests were performed to check the presence of correlations among explanatory factors. We computed the variance inflation factor (VIF) for each predictor variable by doing a linear regression of each predictor on all the other predictors, in each case we obtained VIF within the range of recommended cut of points [26]. The intraclass correlation coefficient (ICC) was computed for each model to show the amount of variations explained at each level of modeling. Model comparisons were done using the Likelihood Ratio Test (LRT), Akaike information criteria (AIC), and Bayesian Information Criteria (BIC). The model with the lowest LRT, AIC, and BIC, was considered the best fit model.

### Ethics approval and consent to participate

The data for this study was received from the DHS office upon request. The data was collected by the Ethiopian Central Statistical Agency (CSA) & the Federal Ministry of Health (FMoH) with the technical assistance of ICF through the DHS Program. The ethical clearance was provided by the Federal Democratic Republic of Ethiopia Ministry of Science and Technology and the Institutional Review Board of ICF International. Written consent to participate in the study was obtained from parents to take blood from children under five for the hemoglobin test, and the data were recorded anonymously.

## Results

### Sociodemographic characteristics of respondents/households

A total of 19,699 mother-child pairs were included in our analysis, 21.8% of the mothers were less than 24 years of age, and 95.1% of the women were in a marital relationship. Nearly, seven in ten women have no formal education, only 4.4% of them have a secondary and higher educational level.

About 89.9% of respondents were rural residents; 22.9% and 22.6% of the HHs were in the range of poorest and poorer wealth quantile respectively, and only 13.7% of HHs were in the richest range. Nearly, 90% of the HHs use agricultural, wood, and animal dung products as a source of cooking fuel, only 1.4% of the HHs had access to electricity and gas (Table 1).

**Table 1:** Sociodemographic characteristics of respondents/households.

Background characteristics	Category	Weighted frequency & Percentage	
		Frequency	Percentage
Age of mother	15–24	4659	21.8
	25–29	6612	30.9
	30–34	4681	21.9
	35–49	5463	25.5
Marital status of women	Never Married	452	2.1
	Married	20367	95.1
	Windowed/divorced/separated	596	2.8
The education level of the mother	No education	15090	70.5
	Primary	5382	25.1
	Secondary	650	3.0
	Higher	294	1.4
Father’s educational level	No education	10547	50.5
	Primary educ	8268	39.6
	Secondary educ	1353	6.5
	Higher educ	726	3.5
Number of under-five children in HHs	1 to 2	17734	82.8
	3 and above	3680	17.2
Number of HH size	2 to 4	5050	23.6
	5 to 7	11089	51.8
	8 and above	5275	24.6
Religion	Orthodox	8167	38.1
	Catholic	227	1.1
	Protestant	4890	22.8
	Muslim	7861	36.7
	Traditional	270	1.3
Media exposure	No	12011	56.1
	Yes	9404	43.9
Place of residence	Rural	19256	89.9
	Urban	2159	10.1
Wealth index	Poorest	4913	22.9
	Poorer	4849	22.6
	Middle	4583	21.4
	Richer	4138	19.4
	Richest	2931	13.7
Cooking fuel	Electricity, and gas	289	1.4
	Fossil fuel	79	.4
	Charcoal	1913	8.9
	Agricultural/wood/animal dung	19134	89.3
Sources of drinking water	Unimproved water source	15255	71.2
	Improved water source	6160	28.8
Toilet facility	Unimproved toilet facility	19792	92.4
	Improved toilet facility	1623	7.6

### Sociodemographic, birth history and physical measurements of under-five children in Ethiopia, EDHS 2005–2016

Of the total children included in the study, 11.9% were 6 to 11 months, 21.2% were 6 to 23 months, and the remaining 66.9% were 24 to 59 months of age. About 18.1% and 8.9% of children were very small, and small at birth respectively, and 17.7% and 33.8% of children were first births, and fifth and above births orders in a family respectively. The finding revealed that 16.8%, 14.6%, and 20.2% of children were reported fever, diarrhea, and cough in the last two weeks before the survey respectively. In the anthropometric measurements, 40.1% of children were stunted, 35.3% were underweight, and 9.2% were wasted (Table 2).

**Table 2:** Sociodemographic, birth history and physical measurements of under-five children in Ethiopia, EDHS 2005–2016.

Background characteristics	Categories	Weighted frequencies & percentages	
		Frequency	Percentages
Sex	Male	10983	51.3
	Female	10431	48.7
Age	6–11 months	2543	11.8
	12–23 months	4553	21.3
	24–59 months	14319	66.9
Child is twin	No	20987	98.0
	Yes	428	2.0
Birth Size	Very small	3866	18.1
	Small	1916	8.9
	Average and larger	15633	73.0
Birth Order	First	3780	17.7
	Second	3525	16.5
	Three to fourth	6870	32.1
	Five and above	7240	33.7
Preceding birth interval	Less than 24 months	3682	17.2
	24 to 47 months	10147	47.4
	More than 48 months	3887	18.1
	First birth	3699	17.3
Succeeding birth interval	Last	14126	66.0
	Less than 24 months	2417	11.2
	More than 24 months	4872	22.8
Fever in the last two weeks	No	17807	83.2
	Yes	3608	16.8
Diarrhea in the last two weeks	No	18279	85.4
	Yes	3136	14.6
Cough in the last two weeks	No	17078	79.6
	Yes	4329	20.2
Stunting	No	12737	59.7
	Yes	8589	40.3
Underweight	No	13762	64.5
	Yes	7564	35.3
Wasting	No	19383	90.8
	Yes	1970	9.2

### The prevalence of anemia among under-five children by different background characteristics

**Fig 1:**
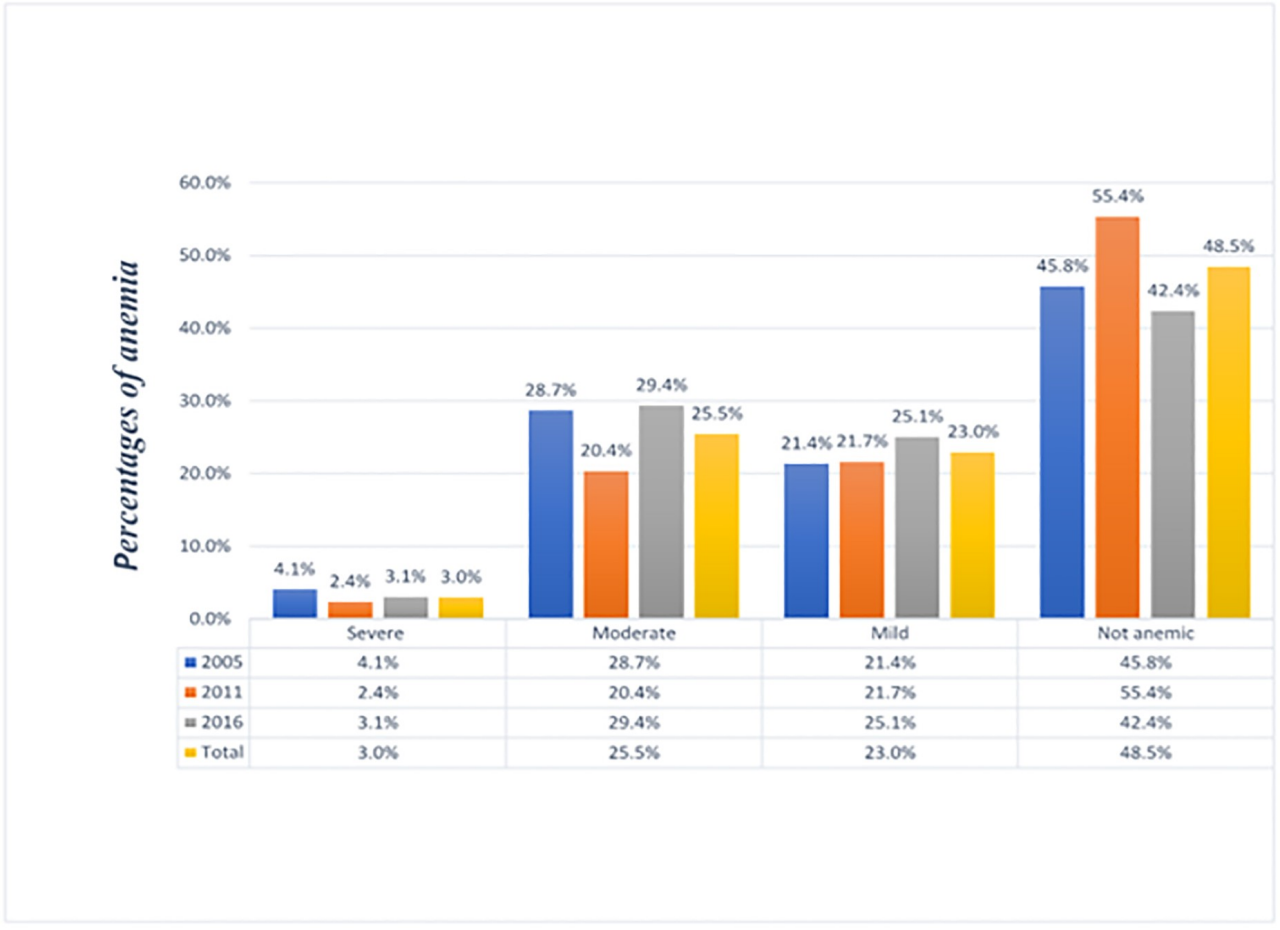
The weighted levels of anemia among children younger than five years in Ethiopia from 2005 to 2016.

The overall prevalence of anemia was 51.5% (95%CI: 50.8–52.2). Of the total 51.5% of the anemia cases, 3%, 25%, and 23% were severe, moderate, and mild cases respectively (Fig 1). The magnitude of anemia across the wave of the EDHS surveys was 55.2% (95%CI: 52.6–55.6.0) in 2005, 44.6% (95%CI: 43.6–45.6) in 2011, and 57.6% (95%CI: 56.5–58.7) in 2016.

Children from HHs utilizing agricultural products & animal dung as a source of cooking fuel showed the highest proportion of anemia (46.2%), only the remaining 5.2% of the cases were from HHs that use other sources of cooking fuels. In addition, the prevalence of anemia from HHs using unimproved water sources and unimproved toilet facilities was 37.3% and 48% respectively (Table 3).

**Table 3:** The weighted prevalence of anemia among under-five children in Ethiopia by different background characteristics, EDHS 2005–2016.

Background characteristics	Anemia status			P-value
	No	Yes	Chi-square	
Place of residence				
Urban	5.8%	4.3%	74.5	<0.001
Rural	42.7%	47.2%		
Wealth index				
Poorest	9.5%	13.5%	201.1	<0.001
Poorer	10.5%	12.1%		
Middle	10.8%	10.6%		
Richer	9.9%	9.4%		
Richest	7.7%	6.0%		
Sources of drinking water				
Unimproved water source	34%	37.3%	13.7	<0.001
Improved water source	14.5%	14.2%		
Cooking fuel				
Electricity and gas	0.72%	0.64%	9.6	0.022
Fossil fuels	0.22%	0.1%		
Charcoal	4.5%	4.5%		
Agricultural products & animal dung	43.1%	46.2%		
Type of toilet facility				
Unimproved toilet facility	44.5%	48.0%	14.4	<0.001
Improved toilet facility	4.0%	3.6%		
Number of under-five children in HH				
One to two	40.9%	41.9%	32.2	<0.001
Three and above	7.6%	9.6%		
Household (HH) size				
Two to four	11.5%	12.0%	.59	0.7
Five to seven	25.1%	26.7%		
Eight & above	11.9%	12.8%		
Total	48.5%	51.5%		

### Regional variations of anemia among under-five children in Ethiopia, EDHS 2005–2016

The findings of this study indicated that children from Somalia regional state were severely affected by anemia, where the prevalence was 86.2% in 2005, 69.6% in 2011, and 83.3% in 2016. The Afar regional state and Dire Dawa city administrations were the second most affected regions; anemia was sustainably higher than 56% across EDHS surveys. Similarly, anemia among children from Oromia, Gambella, and Hareri regions ranges between 50% to 69% across the waves of EDHS surveys.

According to results obtained, a general declining trend of anemia was observed in Tigray, Amhara, Benishangul, and SNNP regions over the years. In addition, the lowest prevalence of anemia was observed among children in Addis Ababa (40% in 2005, 33.1% in 2011, and 48.8% in 2016) (Table 4).

**Table 4:** The weighted frequency and percentage distribution of anemia among children under-five across regions in Ethiopia, EDHS 2005–2016.

Regions	Anemia status					
	2005 N = 3938		2011 N = 8995		2016 N = 8481	
	No	Yes	No	Yes	No	Yes
Tigray	43.1%	56.9%	62.3%	37.7%	46.1%	53.9%
Afar	43.8%	56.3%	25.3%	74.7%	25.3%	74.7%
Amhara	47.9%	52.1%	64.4%	35.6%	57.3%	42.7%
Oromia	43.4%	56.6%	47.9%	52.1%	34.3%	65.7%
Somalia	13.8%	86.2%	30.4%	69.6%	16.7%	83.3%
Benishangul	44.7%	55.3%	53.4%	46.6%	56.7%	43.3%
SNNP	53.0%	47.0%	62.9%	37.1%	49.1%	50.9%
Gambella	40.0%	60.0%	50.0%	50.0%	42.1%	57.9%
Hareri	42.9%	57.1%	44.4%	55.6%	31.3%	68.8%
Addis	60.0%	40.0%	66.9%	33.1%	51.2%	48.8%
Dire Dawa	38.5%	61.5%	37.0%	63.0%	28.1%	71.9%
Total	45.8%	54.2%	55.4%	44.6%	42.4%	57.6%

**Keys**: ‘N’ stands for sample size in each year.

### Results of the geospatial analysis

**Fig 2:**
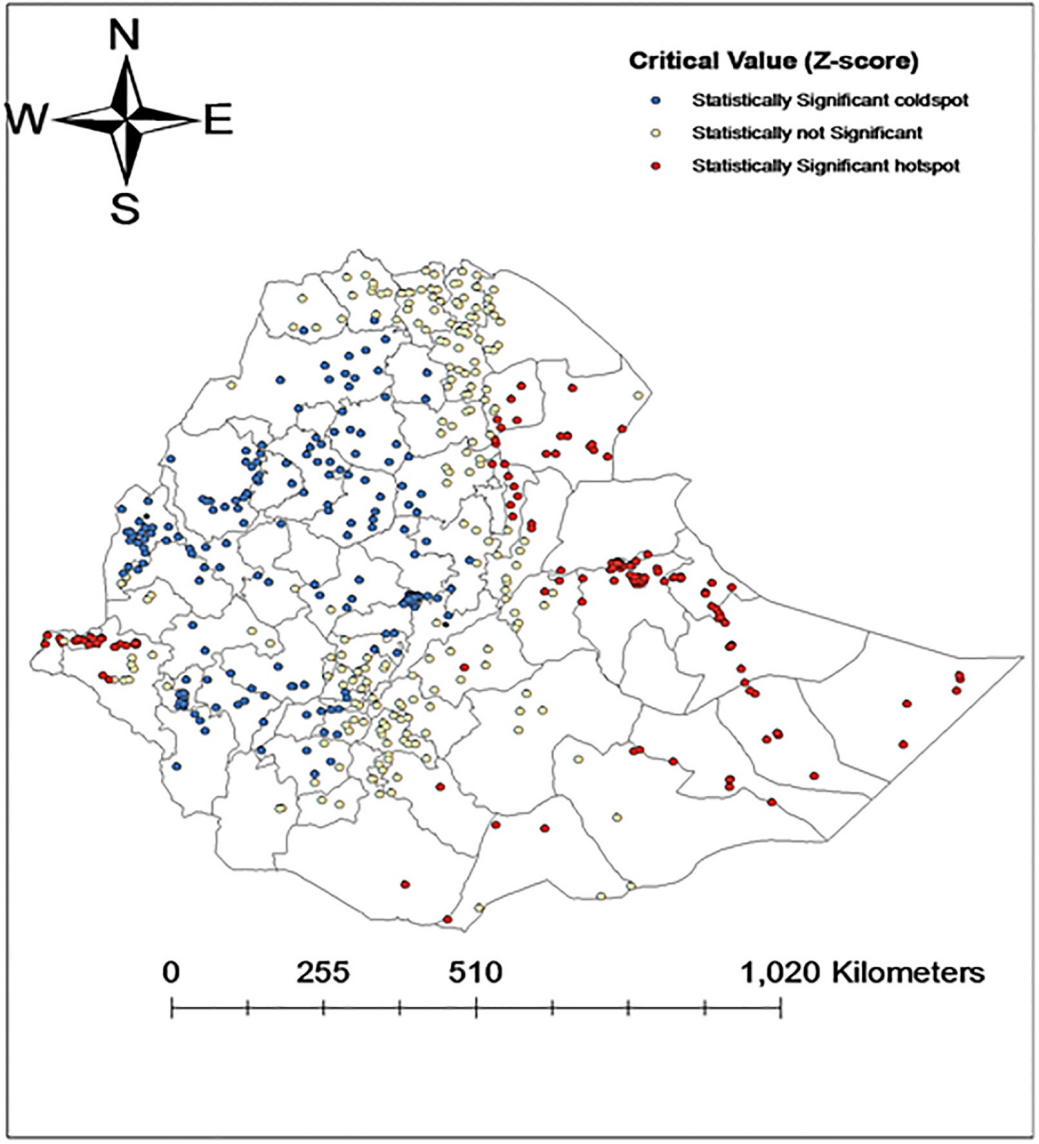
Maps of high and low hotspot clusters of anemia among under-five children in Ethiopia, 2016. Reprinted from [25] under a CC BY license, with permission from [DHS], original copyright [2016].

In the EDHS 2016 cluster-level (lower level) analysis, the Getis-Ord- General statistic tool indicated that the general patterns of anemia distribution among children was not similar across the clusters (Z-score of 2.76, and p-value of 0.006). The hotspots analysis using Getis-Ord- G* statistic showed that high hotspots of anemia were observed in Eastern regions (Somalia, Haregie, and Dire Dawa), Northeastern (Afar region), and Western (Gambella) and Southern parts of the country (few clusters of Oromia region). However, low hotspots were seen in most of the central regions (Addis Ababa, some Oromia zones) and Northwestern (Amhara and Benishangul) regions (Fig 2).

**Fig 3:**
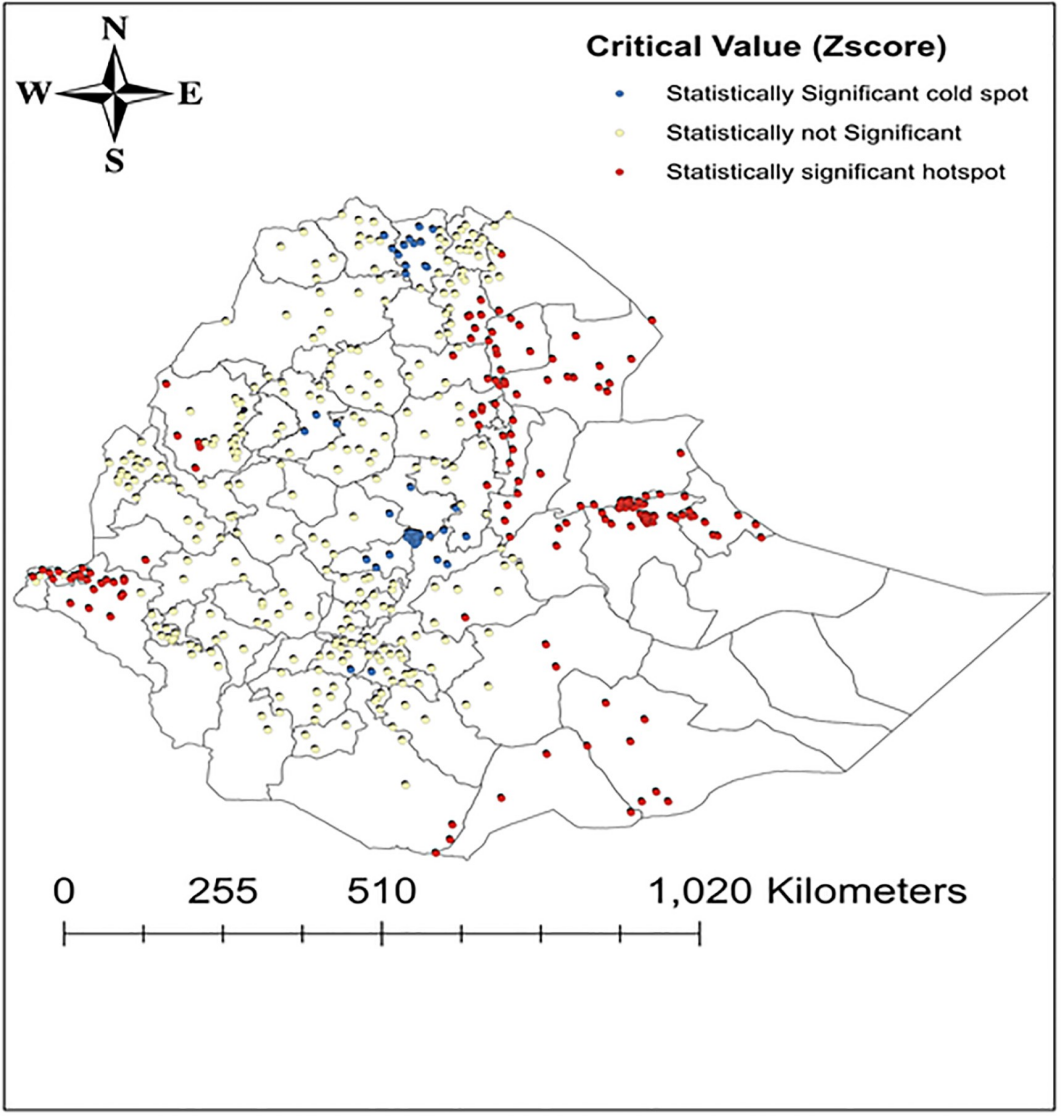
Maps of high and low hotspot clusters of anemia among under-five children in Ethiopia, 2011. Reprinted from [25] under a CC BY license, with permission from [DHS], original copyright [2011].

In the EDHS 2011 spatial analysis, the Getis-Ord- General statistic produced a Z-score of 1.87, and a corresponding p-value of 0.023 showing the overall all patterns of anemia distribution varies across the clusters. The Getis-Ord- G* hotspots analysis revealed that high hotspots of anemia were located in Eastern regions (Harergie, Dire dawa), Southern region (Oromia zones), Northeastern (Amhara and Afar regions), few clusters in North Shewa and Tigray regions, whereas low hotspots have occurred in the central regions (Fig 3).

**Fig 4:**
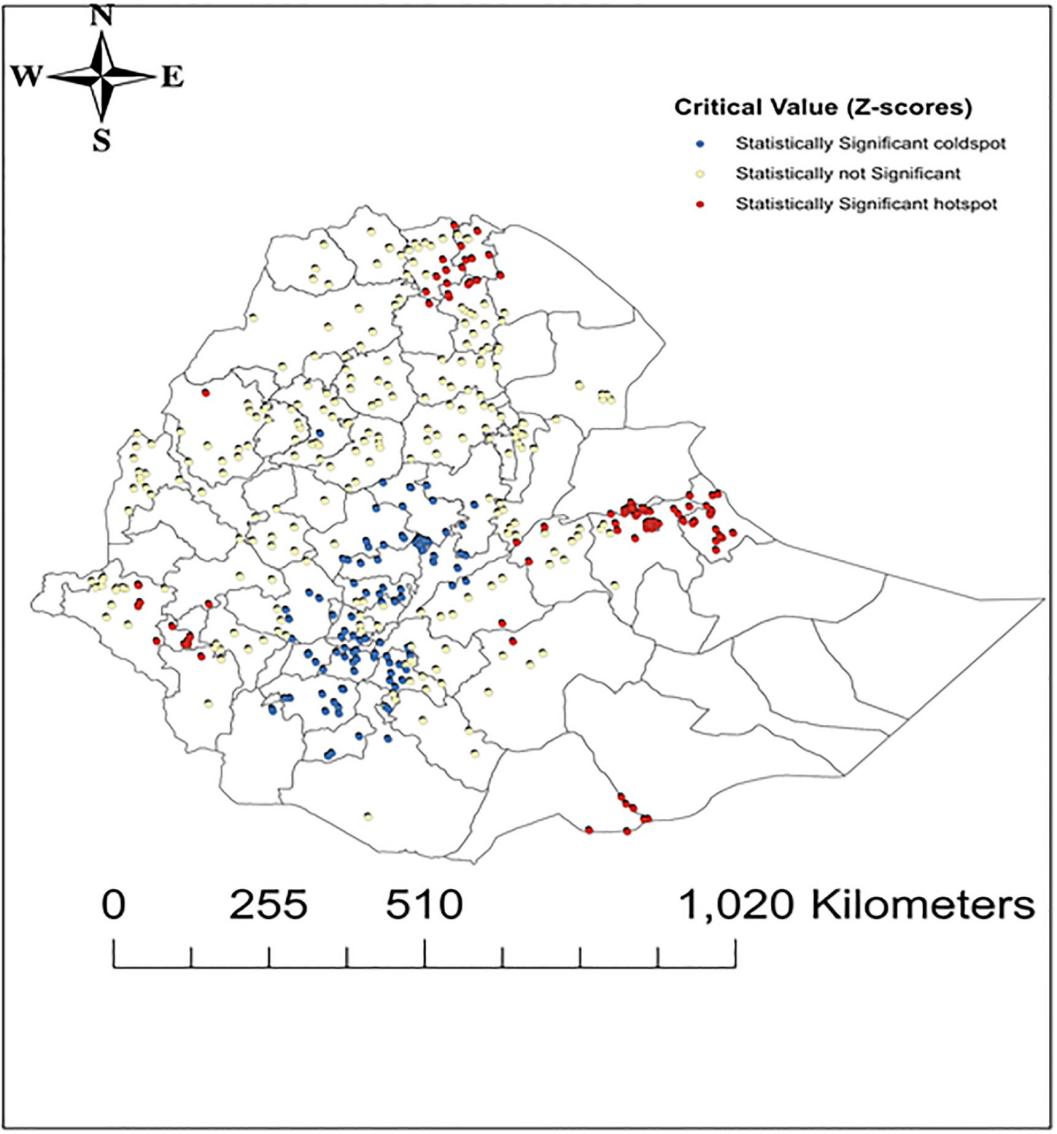
Maps of high and low hotspot clusters of anemia among under-five children in Ethiopia, 2005. Reprinted from [25] under a CC BY license, with permission from [DHS], original copyright [2005].

Similarly, in the 2005 EDHS spatial analysis, the general distribution of anemia was not similar for the clusters (Getis-Ord- General produced Z-score of 2.81 and p-value of 0.0049). The hotspots analysis of Getis-Ord- G* indicated high hotspots of anemia in the Eastern part of the country (Hareri region, Dire Dawa, and few clusters in Somalia regions), and Southern borders of the country and low spots occurred in Addis Ababa (Fig 4).

The legend, critical Z scores grouped into three classes for hotspots graphs for convenience of interpretation in the following ways; Statistically Significant coldspot (Z scores less than -1.96), not Significant (Z scores between -1.96 and +1.96) and Significant hotspot (Z scores greater than +1.96).

### Determinants of anemia among under-five children in Ethiopia, EDHS 2005–2016

A univariate regression analysis was performed to have an insight into the association between anemia and predictor variables. Age, place of residence, maternal educational level, wealth index, birth order, birth intervals, fever, diarrhea, birth size, nutritional status of children, age of mother, number of under-five children in the HHs, household size, media exposure of mother, and years of the survey were showed association with anemia at 20% level of significance (Table 5).

**Table 5:** The unadjusted association between predictor variables and anemia in under-five children in Ethiopia, EDHS 2005–2016.

Background characteristics	Categories	COR & 95%CI for anemia		P-value
		COR	95%CI	
Age of children	6–11 months	3.02	2.73–3.33	<0.001
	12–23 months	2.33	2.17–2.51	
	24–59 months	1.00	1.00	
Place of residence	Urban	1.00	1.00	<0.001
	Rural	1.35	1.25–1.46	
Mother’s educ. Level	No education	1.78	1.45–2.18	<0.001
	Primary educ	1.43	1.16–1.77	
	Secondary educ	1.27	0.99–1.61	
	Higher educ	1.00	1.00	
Marital status of women	Single	0.78	0.61–0.99	0.002
	Married	1.08	0.92–1.28	
	Divorced/widowed	1.00	1.00	
Wealth index	Poorest	1.95	1.79–2.12	<0.001
	Poorer	1.36	1.24–1.49	
	Middle	1.11	1.01–1.21	
	Richer	1.10	0.99–1.21	
	Richest	1.00	1.00	
Birth order	First	1.00	1.00	0.001
	Second	1.03	0.94–1.13	
	Three to fourth	1.23	1.13–1.14	
	Five and above	1.26	1.16–1.36	
Preceding birth interval	Less than 24	1.40	1.28–1.54	<0.001
	24 to 47	1.20	1.11–1.30	
	More than 48	0.97	0.88–1.06	
	First birth	1.00	1.00	
Succeeding Birth Interval	Last	1.54	1.43–1.65	0.001
	Less than 24	1.28	1.16–1.41	
	More than 24	1.00	1.00	
Birth size	Very small	1.27	1.18–1.36	<0.001
	Small	1.17	1.06–1.29	
	Average & larger	1.00	1.00	
Had Diarrhea	No	1.0	1.00	0.001
	Yes	1.32	1.22–1.43	
Had Fever	No	1.00	1.00	<0.001
	Yes	1.23	1.14–1.33	
Stunting	No	1.00	1.00	<0.001
	Yes	1.27	1.19–1.35	
Underweight	No	1.00	1.00	<0.001
	Yes	1.46	1.37–1.55	
Wasting	No	1.00	1.00	<0.001
	Yes	1.69	1.54–1.86	
Number of under-five children in the HHs	1 to 2	1.00	1.00	<.001
	3 and above	1.36	1.27–1.47	
Age of mother	15–24	1.28	1.18–1.39	<0.001
	25–29	1.10	1.02–1.19	
	30–34	1.17	1.08–1.27	
	35–49	1.00	1.00	
Number of HH size	2 to 4	1.09	1.02–1.17	0.009
	5 to 7	1.13	1.04–1.22	
	8 and above	1.00	1.00	
Media exposure	No	1.42	1.34–1.50	<0.001
	Yes	1.00	1.00	
Years of survey	2005	1.00	1.00	<0.001
	2011	0.82	0.75–0.89	
	2016	1.22	1.12–1.32	

### Generalized linear mixed model regression analysis with an intercept only model (Model I)

In model 1, the cluster level regional variation of anemia among children was assessed without considering the effect of individual, household, and contextual level factors. There is statistically a significant variation between clusters in the prevalence of anemia (p-value <0.001). Cluster level variance was 0.253416, and the intraclass correlation coefficient (ICC) between clusters was 0.0715. This indicates that 7.15% of variations in the prevalence of anemia can be explained by clusters (higher level) factors, and the remaining 92.85% of the total variations of anemia is explained within-cluster lower-level units (Table 6).

**Table 6:** Multilevel logistic regression model without explanatory variables (model I).

Fixed part	Coef.	Std. Error	Z	P-value	[95% Cof.Interval]	
*βo*−*intercept*	0.1921914	0.0251806	7.63	.000	0.1428383	0.2415445
Random part	Estimate	Std. Error	[95% Conf. Interval]			
Var(Intercept)	0.253416	.0236821	0.2110026	0.3043549		
Level	ICC	Std. Err.	[95% Conf. Interval]			
Cluster	0.0715201	0.0062057	0.0602715	0.0846789		

### Random intercept and fixed slope GLMM regression for predictors of anemia among under-five children in Ethiopia, EDHS 2005–2016

In the random intercept model (Model II), the effect of individual-level factors on childhood anemia was assessed. The variance of a random factor was 0.2531076 with its standard error 0.0243827 and the confidence level doesn’t include 0.

The age of children was a strong predictor of anemia, children between 6 to 11 and 12 to 23 months had more than three- and two-fold times likely to be anemic compared to children over 24 months (AOR = 3.44, 95%CI: 3.07–3.85, & AOR = 2.40, 95%CI: 2.20–2.61) respectively. Birth order and birth intervals were associated with the presence of anemia. Children with first and second birth orders in a family were less likely to develop anemia compared to those with fifth and above birth orders (AOR = 0.58, 95%CI: 0.37–0.92, & AOR = 0.79, 95%C: 0.71–0.88). The odds of anemia among children whose succeeding birth interval less than 24 months was more than 20% higher compared to those with more intervals (AOR = 1.22, 95%CI: 1.09–1.36).

Children who reported fever recently had higher odds of anemia compared to those with no fever (AOR = 1.12, 95%CI: 1.02–1.22). Stunted and underweight children had more than 25% and 26% likely to develop anemia compared to their counterparts with normal anthropometric measures respectively.

In addition, children from mothers of age 15 to 24 months had more than 37% higher odds of anemia compared to those from mothers of age 35 to 49 years (AOR = 1.38, 95%CI: 1.22–1.56). Eventually, children from mothers who have no access to media exposure were more likely to report anemia compared to those from mothers with access to media (AOR = 1.32, 95%CI: 1.24–1.41 (Model II, Table 7).

**Table 7:** Random intercept, and random coefficient multilevel logistic regression model for predictors of anemia among under-five children in Ethiopia EDHS 2005 to 2016.

		Model II				Model III			
Variables	Categories	P-value	AOR	[95%CI AOR]		P-value	AOR	[95%CI AOR]	
				Lower	Upper			Lower	Upper
Age in months	6–11	<0.001*	3.35	2.99	3.76	<0.001*	3.44	3.07	3.85
	12–23	<0.001*	2.34	2.15	2.55	<0.001*	2.40	2.20	2.61
	24–59	.	.	.	.	.	.	.	.
Mother’s educ. level	No education	0.09	1.24	0.97	1.57	0.19	1.17	0.93	1.47
	Primary educ	0.30	1.14	0.89	1.44	0.69	1.05	0.83	1.32
	Secondary educ	0.50	1.09	0.84	1.42	0.54	1.09	0.84	1.41
	Higher educ	.	.	.	.	.	.	.	.
Birth order	First	0.030*	0.60	0.38	0.95	0.02*	0.58	0.37	0.92
	Second	0.002*	0.83	0.73	0.93	<0.001*	0.79	0.71	0.88
	Three- fourth	0.330	0.96	0.88	1.04	0.21	0.95	0.87	1.03
	Five & above	.	.	.	.	.	.	.	.
Preceding Birth interval in months	< 24	0.45	0.83	0.52	1.34	0.69	0.91	0.57	1.45
	24 to 47	0.26	0.76	0.47	1.22	0.33	0.79	0.50	1.26
	> 48	0.11	0.68	0.42	1.09	0.12	0.69	0.43	1.10
	First birth	.	.	.	.	.	.	.	.
Succeeding Birth Interval in months	Last	0.004*	1.14	1.04	1.24	0.06	1.08	0.99	1.17
	< 24	0.014*	1.15	1.03	1.28	<0.001*	1.22	1.09	1.36
	> 24	.	.	.	.	.	.	.	.
Birth size	Very small	0.048*	1.09	1.01	1.18	0.07	1.08	0.99	1.17
	Small	0.61	1.03	0.92	1.15	0.42	1.05	0.94	1.16
	Average & larger	.	.	.	.	.	.	.	.
Diarrhea	Yes	0.44	1.04	0.95	1.14	0.70	1.02	0.93	1.12
	No	.	.	.	.	.	.	.	.
Fever	Yes	0.003*	1.14	1.04	1.24	0.012*	1.12	1.02	1.22
	No	.	.	.	.	.	.	.	.
Stunting	Yes	<0.001*	1.26	1.17	1.37	<0.001*	1.25	1.16	1.35
	No	.	.	.	.	.	.	.	.
Underweight	Yes	<0.001*	1.26	1.16	1.37	<0.001*	1.26	1.16	1.37
	No	.	.	.	.	.	.	.	.
Wasting	Yes	0.008*	1.16	1.04	1.30	0.002*	1.19	1.07	1.33
	No	.	.	.	.	.	.	.	.
Age of mother	15–24	<0.001*	1.37	1.20	1.55	<0.001*	1.38	1.22	1.56
	25–29	0.002*	1.17	1.06	1.29	0.003*	1.16	1.05	1.27
	30–34	0.005*	1.14	1.04	1.25	0.004*	1.14	1.05	1.25
	35–49	.	.	.	.	.	.	.	.
Media exposure	No	0.012*	1.10	1.02	1.18	<0.001*	1.32	1.24	1.41
	Yes	.	.	.	.	.	.	.	.
Wealth index	Poorest	.	.	.	.	<0.001*	1.67	1.45	1.93
	Poorer	.	.	.	.	0.003*	1.25	1.08	1.45
	Middle	.	.	.	.	0.75	1.02	0.89	1.19
	Richer	.	.	.	.	0.22	1.09	0.95	1.26
	Richest	.	.	.	.	.	.	.	.
NO of HHs size	2 to 4	.	.	.	.	0.34	0.95	0.84	1.06
	5 to 7	.	.	.	.	0.96	1.01	0.92	1.09
	8 & above	.	.	.	.	.	.	.	.
U5 children in the HHs	1 to 2	.	.	.	.	<0.001*	0.83	0.76	0.91
	3 and above	.	.	.	.	.	.	.	.
Residence	Urban	.	.	.	.	0.17	0.91	0.79	1.04
	Rural	.	.	.	.	.	.	.	.
Year	2005	.	.	.	.	<0.001*	0.82	0.75	0.90
	2011	.	.	.	.	<0.001*	0.65	0.60	0.69
	2016	.	.	.	.	.	.	.	.
**Random part Model II**						**Random part model III**			
Random Effect	Estimate		[95%CI for Var]			Estimate	[95%CI for var]		
Var (Intercept)	0.2531076		0.2095589	0.3057062		0.2387039	0.1967381		0.2896214
Level (EA’s)	ICC		[95%CI for ICC]			ICC	[95%CI for ICC]		
	0.0714393		0.0598838	0.0850229		0.0676489	0.0564268		0.0809114

**Keys**: Var = variance, ICC = interclass correlation coefficient, NO = Number of, U5 = under five

* = indicates significant.

### Random intercept and coefficient multilevel logistic regression model for predictors of anemia among under-five children in Ethiopia, EDHS 2005–2016

The random coefficient (Model III) consists of both individual and contextual level variables in addition to cluster-specific random effects. The ICC was 0.0676 and variance was 0.2387039, with a standard error of.0235482, and 95% CI doesn’t contain 0, indicating that variation is significant.

The odds of anemia was higher among children between 6 to 11 and 12 to 23 months compared to children above two years (AOR = 3.35, 95%CI: 2.99–3.76, & AOR = 2.34, 95%CI: 2.15–2.55) respectively. Children with first and second in the birth order were 40% and 17% less likely to be anemic compared to those with 5 or above birth order (AOR = 0.60, 95%CI: 0.38–0.95, & AOR = 0.83, 95%CI: 0.73–0.93) respectively.

Children reported fever, and children with low height and weight for the age on anthropometric measurement were more likely to be anemic compared to their counterparts with no fever, and normal anthropometric measurement respectively. The finding also showed that as the age of a mother increases the odds of anemia in children decreases, and children from a mother with no access to media were more likely to be anemic compared to children from mothers with access to media.

Children from HHs with the poorest and poorer wealth quantile were more likely to be anemic compared to those the richest quantile (AOR = 1.672, 95%CI: 1.450–1.927, & AOR = 1.249, 95%CI: 1.079–1.445) respectively. Besides, the number of under-five children in the HHs has an effect on anemia, children from HHs with one to two number of children in a family had more than 17% less chance of getting anemia compared to children from HHs with three and more children (AOR = 0.830, 95%CI: 0.755–0.913) (model III, Table 7).

### The goodness of fit of models in multilevel logistic regression

The appropriateness, adequacy, and usefulness of the models were tested using AIC, BIC, and Likely hood Ratio Test (LRT) tests. The empty model has the highest Deviance, LRT, and AIC; however, it is statistically significant indicating that a model with a random intercept is better than a model without a random intercept. The random coefficient model has the lowest AIC, BIC, and LRT values compared to the empty and random intercept models. This shows that the random coefficient model is the best fit model (Table 8).

**Table 8:** Multilevel logistic regression analysis on the prevalence of anemia among under-five children, model comparisons.

	Empty model (Model I)	Random intercept model (Model II)	Random coefficient model (Model III)
ICC	0.0715201	.0714393	0.0676489
-2*loglikelihood	26626.384	24999.59	24699.902
LRT	479.84	421.44	382.00
P-value	0.000	0.000	0.000
AIC	26630.38	25051.59	24771.9
BIC	26646.16	25256.48	25055.6
DF	2	26	36

## Discussions

According to the WHO criteria, anemia is highly prevalent and considered a severe public health significance (defined as a prevalence higher than 40%) in most middle- and low-income countries [27]. Severe anemia has an increased risk of mortality and negative long-term consequences of damaging cognitive performance and motor development in children. Subsequently, this can result in impaired economic productivity and developments in the nations [28–30].

The Sustainable Development Goal (SDGs) addresses anemia indirectly, the second goal is about ending hunger, aims to end all forms of malnutrition by 2030. In particular, the goal focuses to address the nutritional requirements of under-five children, adolescent girls, and mothers [31].

Despite the availability of integrated community-based child health care and various children focused prevention and intervention programs to avert nutritional disorders and childhood diseases in Ethiopia [32–34], the findings of this study showed that the prevalence of anemia across the wave of the EDHS surveys was unacceptably higher.

The magnitude was 54.2% in 2005, and showed an insubstantial declining trend into 44.6% in 2011 but increased back to 57.6% in 2016. The finding also showed that there is a huge disparity in the prevalence of anemia across the country in each survey. Somalia regional state was the most affected region, the prevalence was 86.2% in 2005, 69.6% in 2011, and 83.3% in 2016. Afar regional state and Dire Dawa city administration were the second most affected regions, anemia higher than 56% in all of EDHS surveys between 2005 to 2016. The geospatial analysis supports these findings that most of the hotspots areas of anemia were located in the East, Northeast, and Western regions of the country, however, most of the low hotspot areas of anemia were located in the Central region, and South, Northwest and Northern parts of the country (Figs 2–4). The observed regional variability of anemia could be attributed to the regional differences in child feeding habits, infectious disease distributions, and availability and access to health care services [35–37].

The age of children was a strong predictor of anemia both in the random intercept (Model 2) and random slope (Model 3). The odds of anemia decreases as the age of children increases; children whose age ranges within 6 to 11 months, and 12 to 23 months had more than three and two-fold risks of anemia compared to those with over 24 months respectively. This finding hasn’t supported the evidence that absorbed iron requirements increases with age, similar to other energy requirements in children [38], however, children above the age of one year commonly consume a variety of food sources rich in iron contents including meats, poultry, fish, cereals [39, 40]. In addition, the nutritional disorder is much higher in younger than older age children [41, 42]. Moreover, younger children are highly vulnerable to infectious diseases such as intestinal helminths as they might ingest contaminated materials into their mouths compared to older ones, particularly children living in an unsanitary environment [43, 44]. This finding is supported by similar other studies conducted across the globe that children less than two years of age were at higher risk of anemia [10, 11, 15].

In this study, children with first and second birth orders in a family were less likely to be anemic compared to those with fifth or above in the birth order. This could be an increase in the number of children associated with increased health problems due to competition for food, infections, and cross contaminations [45–47]. This finding is supported by studies conducted in Uganda and Cameroon, the number of children in a family significantly associated with anemia in children [11, 48].

Birth interval showed an effect on anemia status, children with birth interval less than 24 months before their younger siblings were at higher risk of anemia compared to those with optimal intervals. This could be attributed to maternal nutritional depletion, vertical transmission of infections, suboptimal lactation due to pregnancy overlap, sibling competition for food, and transmission of infectious diseases among siblings [49]. Our finding is in line with studies conducted in African countries where birth spacing is associated with anemia in children [50, 51].

This study also showed that the odds of anemia was much higher among children who had a fever recently compared to those with no symptom. Even though the underlying cause of fever may be different, it could be due to systemic infections in the body that might affect the hemoglobin level in the blood [52, 53]. Evidence showed that fever can happen due to malaria or any other disease in a situation where both fever and anemia coexist [54, 55]. Similar studies conducted recently in Sub-Saharan African countries revealed that children who had a fever recently were more likely to be anemic compared to those with no fever [15, 56].

In this study, the odds of anemia among children with low height and weight for age on anthropometric measurements was much higher compared to those with normal anthropometric measurements. This might be due to the nutritional status of children directly affects the hemoglobin level in the blood [57, 58]; several studies conducted so far across the globe showed that stunting and underweight among children were associated with anemia [11, 58–63].

Maternal age showed an effect with anemia status of their children, mothers whose age ranges between 15 to 24 years had more than 36% higher risk of anemia compared to those between 35 to 49 years. This could be due to low maternal age contributes to low birth weight (LBW) babies [64–66], in turn, LBW in children might contribute to low hemoglobin level in the blood [67–69].

This study also indicated that children from mothers with no media exposure had more than 30% higher odds of anemia compared to those from mothers with media exposure. This finding is supported by a study conducted in India, children from mothers with no media exposure were more likely to report anemia compared to their counterparts (63.6% vs. 53.5%) [70]. The reason could be mothers’ media exposure may affect childcare practices through enhancing the knowledge of mothers on child feeding activities, disease prevention practices, and improving health-seeking behaviors [71, 72]. Another study conducted in India showed that women who received health education were more likely to be familiar with anemia prevention practices compared to women who didn’t [73].

Socio-economic status of the households showed association with anemia, children from HHs with poorest and poorer wealth quantile had more than 67% and 45% chance of getting anemia compared to children from richest wealth quantile respectively. This could be due to HHs with higher wealth quintile are more likely to provide balanced macro and micronutrients (minerals and vitamins) to their children, and children from these HHs have more chance of accessing health care services. Several studies confirm that children from a lower economic status are vulnerable to various nutritional disorders including anemia, and at risk of easily preventable diseases [10, 15, 56, 74].

Moreover, the number of under-five children in the HHs has an effect on childhood anemia, children from HHs with two or less number of children had more than 17% less chance of getting anemia as compared to those from HHs with more number of children. This could be due to an increase in the number of children might lead to a risk of communicable disease transmission, and competition for food, consequently, nutritional deficiencies [10, 56]. This finding is supported by similar studies, that the number of children in the HHs associated with anemia [11, 15].

The potential strength of our study is the use of all available data (EDHS 2005 to 2016); this enables us to have a large sample size. In addition, the use of multilevel and geospatial analysis to handle the clustering effect of the data, and to have a better insight to locate the high and low hotspot areas of anemia across the country. Despite its strength, these findings should be interpreted considering its limitations. The temporal relationship between childhood anemia and explanatory factors can’t be established due to the cross-sectional nature of the source data. Exposure variables such as the presence of fever, and diarrhea before the survey were based on women’s self-report that could result in recall bias or varies according to the illness perception of the mother. Moreover, the birth size of children is taken from the subjective report of the mothers, which could have resulted in bias. Nevertheless, these biases are non-differential, as they are independent of the characteristics of women or children.

## Conclusions

The findings of this study indicated that more than one-half of the children (51.5%) were anemic, and the prevalence of anemia sustainably higher than the expected level across the waves of the EDHS surveys from 2005 to 2016. The geospatial distribution of anemia among under-five children significantly varies across regions in Ethiopia; high hotspots of anemia were concentrated in the East, Northeast, and Western regions of the country. However, low hotspots were seen in most of the Central, South, North, and Northwest regions of the country.

The risk of anemia is highest in the first two years of life, therefore, the families, health care workers, and program planners on child health care should emphasize this critical period. Birth order and birth interval of children have a strong association with anemia, and the risk of anemia is much higher for higher-order births, and for whom birth interval is less than 24 months from their younger siblings. This demands actions by the government and concerned organizations to work further on family planning services to limit family size and integrate it with health promotion to have adequate birth spacing between siblings.

Besides, as the wealth index of the HHs improves from poorest to richest, the risk of anemia decreases significantly. Moreover, children from HHs with one or two number of under-five children had less probability of developing anemia compared to children from HHs with more children. Therefore, problem tailored interventions by government, regional health offices, and concerned organizations should work in harmony to avert the consequences of anemia in children.
